# Inflammatory processes involved in NASH-related hepatocellular carcinoma

**DOI:** 10.1042/BSR20221271

**Published:** 2023-01-24

**Authors:** Stefania Cannito, Umberto Dianzani, Maurizio Parola, Emanuele Albano, Salvatore Sutti

**Affiliations:** 1 Department of Clinical and Biological Sciences, Unit of Experimental Medicine and Clinical Pathology, University of Turin, Turin, Italy; 2 Department of Health Sciences and Interdisciplinary Research Centre for Autoimmune Diseases, University of East Piedmont, Novara, Italy

**Keywords:** Hepatocellular carcinoma, Liver inflammation, lymphocytes, Nonalcoholic steatohepatitis

## Abstract

Hepatocellular carcinoma (HCC) is the fourth leading cause of cancer-related death worldwide. In the recent years nonalcoholic fatty liver disease (NAFLD) is becoming a growing cause of HCCs and the incidence of NAFLD-related HCCs is expected to further dramatically increase by the next decade. Chronic inflammation is regarded as the driving force of NAFLD progression and a key factor in hepatic carcinogenesis. Hepatic inflammation in NAFLD results from the persistent stimulation of innate immunity in response to hepatocellular injury and gut dysbiosis as well as by the activation of adaptive immunity. However, the relative roles of innate and adaptive immunity in the processes leading to HCC are still incompletely characterized. This is due to the complex interplay between different liver cell populations, which is also strongly influenced by gut-derived bacterial products, metabolic/nutritional signals. Furthermore, carcinogenic mechanisms in NAFLD/NASH appear to involve the activation of signals mediated by hypoxia inducible factors. This review discusses recent data regarding the contribution of different inflammatory cells to NAFLD-related HCC and their possible impact on patient response to current treatments.

## Introduction

In the last decade nonalcoholic fatty liver disease (NAFLD) has emerged as the most common cause of chronic liver disease worldwide with a global prevalence of about 25%, ranging from 13% in Africa up to 42% in southeast Asia [1]. Current estimates indicate that NAFLD prevalence in Europe, U.S.A. and Asia is going to increase by 2030 affecting more than 400 million individuals [2]. Recently the disease as received a new terminology of metabolic dysfunction-associated fatty liver disease (MAFLD) better focalizing on positive diagnostic criteria related to the presence of insulin resistance, obesity, and Type II diabetes [3]. Liver lesions associated with MAFLD/NAFLD encompasses a spectrum of conditions ranging from simple steatosis to steatohepatitis, so far still named nonalcoholic steatohepatitis (NASH), which combines fatty liver with parenchymal damage (apoptosis and ballooning, focal necrosis, lobular/portal inflammation, and variable degree of fibrosis [4].

While simple steatosis has low risk of further liver complications, NASH can progress to fibrosis/cirrhosis and NASH-associated liver fibrosis is the strongest predictor for disease-specific mortality [4]. A growing matter of concern relates to the increasingly frequent association between NASH and the development of hepatocellular carcinoma (HCC). At present, HCC accounts for most of primary liver malignancies and is the fourth leading cause of cancer-related mortality worldwide being responsible for more than 800,000 deaths annually [5]. At difference of other cancers, in the last decades the rate of death from HCC has increased in many countries, while the 5-year survival rate remains approximately 18% [5]. Although the incidence of MAFLD/NAFLD-related HCC is still lower than that of HCC of other aetiologies, the recent diffusion of effective therapies for hepatic viral infections is changing this scenario. In fact, developed countries are facing an increasing burden of NAFLD-related HCCs, which are now accounting for approximately 1–38% of all HCCs [6], but these figures are expected to increase by 122% within 2030, making MAFLD/NAFLD the prevalent cause of HCC in the next decade [6]. Furthermore, differently from viral HCCs, MAFLD/NAFLD-related HCC occurs in up to 50% of the cases in the absence of cirrhosis [6]. The estimated annual HCC incidence among non-cirrhotic MAFLD/NAFLD patients is lower (0.1–1.3 per 1,000 patient-years) as compared with those with cirrhosis (0.5–2.6% per 1,000 patient-years) [6]. However, the lack of HCC screening protocols in MAFLD/NAFLD patients without cirrhosis contributes to late diagnosis and hampers the treatments of these tumours. These aspects and the fact that MAFLD/NAFLD prevalence is higher than that of other liver diseases requires urgent measures to control the risk factors involved in the development MAFLD/NAFLD-related HCC [7].

From the histopathologic point of view, NAFLD-related HCCs are often characterized by a specific morphology, known as steatohepatitic HCC, involving the presence of macrovesicular steatosis, cell ballooning along with the presence of Mallory-Denk bodies, inflammation, and variable fibrosis reminiscent of the features of NASH [8]. Furthermore, as compared with HCCs of other aetiologies, these tumours show increased staining for inflammatory markers such as C-reactive protein and serum amyloid A [8].

So far, the mechanisms responsible for the onset and evolution of HCC in NAFLD/NASH are still poorly understood. Epidemiological studies have shown that HCC risk is strictly associated with the prevalence of obesity and Type II diabetes [7,9]. Moreover, among the genetic factors predisposing to MAFLD/NAFLD in the different ethnicities the rs738409 polymorphism in patatin-like phospholipase domain containing 3 (PNPLA3) appears the most common risk factor associated to the disease progression to advanced fibrosis and HCC [7,9]. These data suggest the possibility that the derangement of hepatocyte lipid homeostasis leading to fatty liver and lipotoxicity might represent the cellular background responsible for neoplastic transformation. Nonetheless, a recent metanalysis indicates that the rate of NAFLD progression to HCC increases by over 10 folds in the patients with established NASH as compared with those with simple steatosis [7]. On the same vein, molecular characterization of NAFLD/NASH-associated HCCs has shown that the tumour mutational burden is higher in non-cirrhotic than in cirrhotic HCCs pointing to the possibility that hepatic inflammation could play a major role in the pathogenesis of these specific tumours [10]. Along this line, mice models of NAFLD-related HCCs involves the presence of extensive lobular inflammation and the development of liver cancer is negligible in experimental conditions leading only to simple steatosis [11]. Furthermore, in some of these models such as the choline-deficient high fat (CDHF) diet or the combination of the administration of the liver carcinogen diethylnitrosoamine (DEN) with NASH inducing treatments such as high fat/carbohydrate (HFC) or choline-deficient amino acid sufficient (CDAA) diets, the modulation of hepatic inflammation directly influences HCC development [12–14]. Although chronic inflammation is recognized as a critical step in hepatic carcinogenesis, the exact mechanisms leading to HCC are still incompletely elucidated, while emerging evidence points to anti-tumour activities of immune cells in NASH [15]. In this review, we will discuss in detail the contribution of both innate and adaptive immune cells in hepatic carcinogenesis associated with NAFLD/NASH as well as the possible implication factors modulating the inflammatory environment of NASH livers.

## Inflammatory mechanisms in NASH evolution

**Figure 1:**
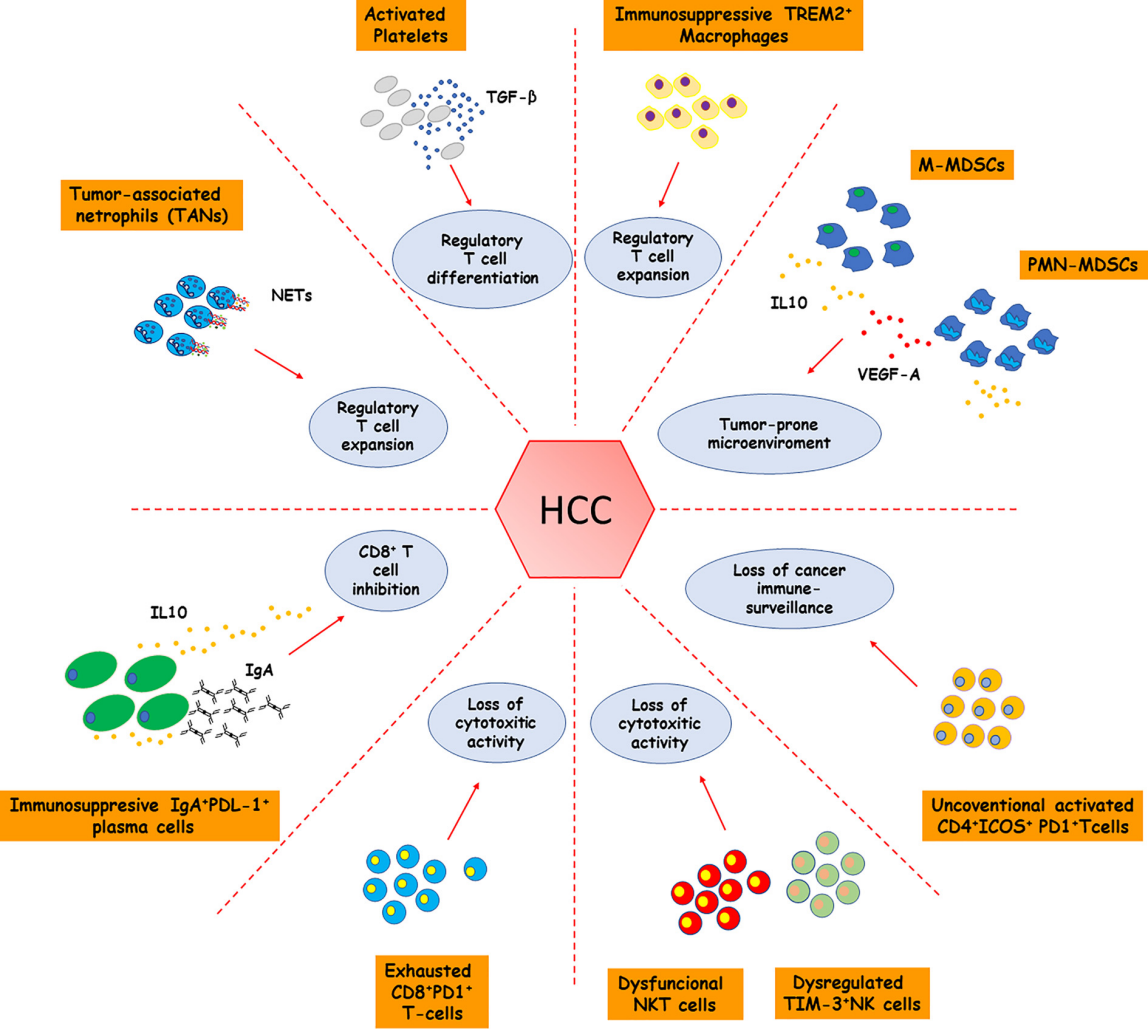
Contribution of immune/inflammatory cells to immune elusion by NASH-related HCC NASH-associated HCC immune landscape is characterized by the expansion of cellular pools displaying potent immunosuppressive activities such as monocytic and polymorphonuclear myeloid-derived suppressor cells (M- and PMN-MDSCs), tumor-associated neutrophils (TANs), activated platelets, immunosuppressive TREM-2^+^ macrophages, regulatory T-cells, and IgA^+^/PDL-1^+^ plasma cells. Overall, these cell subsets contribute to limiting cancer immune surveillance by producing large amounts of immunomodulant mediators such as transforming growth factor-β1 (TGF-β1) and interleukin (IL)-10 that, in their turn, counteract effector T-cell functions. NASH-related HCC stands out for the accumulation of unconventional activated CD4^+^/ICOS^+^/PD-1^+^ T-cells and exhausted CD8^+^/PD1^+^ probably resulting from chronic antigen stimulation involving oxidative stress-derived epitopes (OSEs) and damage- and pathogen-associated molecular patterns (DAMPs and PAMPs) among others. The transition from NASH to HCC is also associated with the progressive development of dysfunctional NK/NKT cells showing impaired cytotoxic activity toward cancer cells.

The transition from simple steatosis that characterizes NAFLD to NASH is a complex process involving multiple factors including metabolic dysfunctions, lipotoxicity, gut dysbiosis, oxidative stress, and hepatocellular necrosis. All these factors stimulate the induction of chronic inflammation responsible for perpetuating tissue injury, parenchymal cell regeneration, mutagenesis, and HCC progression [16]. The liver contains a variety of immune cells that in physiological conditions contribute to preserve an immunotolerant microenvironment [17]. Hepatic immunotolerance is fundamental for maintaining tissue homeostasis because the liver is continuously exposed to food-derived antigens and bacterial products originating from gut microbiota and reaching the organ through portal circulation [17,18]. In NAFLD livers, the increased influx of free fatty acids (FFAs) leads to lipotoxic injury, oxidative stress, and cell death that along with gut dysbiosis trigger inflammation subverting such an immunotolerant environment [19,20]. Regarding dysbiosis, NAFLD is accompanied by abnormal growth of harmful bacterial strains that induce an increased permeability of the mucosal barrier, known as leaky gut syndrome, thus favoring the translocation of bacterial products to the liver [18]. Interestingly, similar alterations in gut microbiome are also evident in NAFLD-related HCCs [21]. In this scenario, the persistence of cellular damage and influx of pathogen-associated molecular patterns (PAMPs) foster chronic hepatic inflammation leading to fibrosis and cirrhosis, working as a fertile ground for HCC development [15,16]. Chronic inflammation and hepatocyte death also cause hepatic stellate cell (HSC) differentiation to myofibroblasts that represent the main source of extracellular matrix (ECM) components [22]. Chronic tissue loss also fosters cell proliferation which involve both hepatocytes and liver progenitor cells [23], while inflammation promotes the production of reactive oxygen species (ROS) increasing the frequency of DNA damages and mutations [24]. The combination of high cellular proliferation rates and DNA mutations creates ideal conditions for malignant transformation [15]. In these settings, a further contribution in supporting hepatic inflammation might come from platelets since recent reports have described an increased hepatic infiltration by platelets in both humans and mice suffering from NASH [25,26]. NAFLD/NASH patients also display an increased mean platelet volume (MPV), an indicator of high platelet production [27], which correlates with the degree of inflammation and fibrosis [28]. Platelets are rapidly activated following tissue damage, releasing IL1β-loaded microparticles [29] as well as granules containing inflammatory cyto/chemokines and growth factors including tumour necrosis factor α (TNFα), interleukin-6 (IL-6), transforming growth factor β (TGF-β1), platelet-derived growth factor (PDGF), endothelial growth factor (EGF), insulin-like growth factor 1 (IGF-1), vascular endothelial growth factor A (VEGF-A), hepatocyte growth factor (HGF), and fibroblast growth factor (FGF) [30,31]. Consistently, pharmacological inhibition of platelet activation in mice abrogates hepatic immune cell infiltration and prevents NASH-induced HCC development [26]. Noteworthy, platelets may also contribute to tumor progression and metastasis by fostering the differentiation of regulatory T-lymphocytes (Tregs) via TGF-β1 signaling and by releasing pro-angiogenetic factors [32] (Figure 1).

Although current view indicates that innate immune mechanisms represent a key element in supporting hepatic inflammation in NASH, increasing evidence points to an additional role of adaptive immunity in NASH progression to fibrosis and HCC [33]. Histology shows that B- and T-lymphocytes are present in either lobular or periportal infiltrates [34] often forming focal aggregates, resembling ectopic lymphoid structures [35]. Hepatic infiltration by B- and T-lymphocytes is also evident in different experimental models of NASH, where it parallels the worsening of parenchymal injury and lobular inflammation [33]. In these settings, B-lymphocyte response involves the fraction of CD43^−^/CD23^+^ B2-cells, while T-lymphocytes include proinflammatory CD4^+^ interferon-γ (IFN-γ)-producing T-helper 1 (Th-1), IL-17 producing T-helper 17 (Th-17) cells and cytotoxic CD8^+^ T-cells [33;36]. The contribution of adaptive immunity to NASH is further supported by the observations that steatosis, parenchymal injury, and lobular inflammation are lowered in immunocompromised Rag1^−/−^ mice, lacking mature B-, and T-cells [12] as well as following the selective ablation of B-lymphocytes or CD8^+^ T-cells [36,37]. From a mechanistic point of view, the cytokine network generated by Th-1 and Th-17 CD4^+^ may provide a stimulus for the proinflammatory activity of macrophages [33,38], while CD8^+^ T-cells are cytotoxic against hepatocytes through antigen independent mechanisms [36]. On their turn, innate immunity cells support lymphocyte functions through release of interleukin 12 (IL-12), 15 (IL-15) and 23 (IL-23) as well as lymphocyte chemokines CXCL9-10-11 [33,38]. However, chronic inflammation also deeply influences the hepatic immune system allowing cancer cells to escape to the immunosurveillance establishing a microenvironment prone to the tumor growth [39]. Cancer development is in fact associated with macrophage reprogramming from a pro-inflammatory to an immunosuppressive phenotype accompanied by an enrichment of regulatory CD4^+^/FOXp3^+^ Tregs and exhausted CD8^+^-T-lymphocytes [40,41].

## Liver macrophages in the track from NASH to HCC

Inside the liver, the presence of danger signals is sensed through the engagement of pattern recognition receptors (PRRs) expressed on Kupffer cells (KCs) triggering their activation [17] and the secretion of proinflammatory cyto/chemokine such as TNF-α, CCL2, CXCL10 and CXCL8 (IL-8) which promote the recruitment and activation of circulating leukocytes responsible for sustaining hepatic inflammation [42]. Among the recruited cells, a critical role is played by monocytes that inside the liver differentiate into monocyte-derived macrophages (MoMFs) characterized by a pro-inflammatory behavior [42,43]. During the disease evolution, MoMFs significantly contribute to inflammatory response and by surrounding dead/dying-steatotic hepatocytes give rise to aggregates known as lipogranulomas or hepatic crown-like structures (hCLSs) [44–46]. Notably, macrophages forming hCLSs display a peculiar phenotype characterized by the expression of Triggering Receptor Expressed on Myeloid cells 2 (TREM2), CD63 and the glycoproteins CD9 and NMB (GPNMB) [45–47]. Because of their association with NASH these cells have been renamed NASH-associated macrophages (NAMs) [45]. Interestingly, from a phenotypical point of view, NAMs resemble scar-associated macrophages described in human fibrotic livers [48]. On this respect, NAMs produce pro-fibrogenetic mediators such as osteopontin (OPN) and galectin-3 and localize in regions rich in collagen fiber deposition suggesting their possible involvement in NASH-related fibrogenesis [49]. However, in CCR2-knockout mice, the impairment of monocyte recruitment reduces hCLS formation while fosters hepatic fibrosis, suggesting that NAMs might represent a heterogenous population also involved in controlling tissue scarring [46]. At present, several evidence indicate a pro-fibrogenetic role of NAMs [49–51] and points to their involvement in the transition from NASH to HCC since the presence of lipogranulomas associates with HCC development [52]. Indeed, most HCCs develop in the context of cirrhotic regenerative nodules where the reduction of sinusoid porosity along with the collagenization of the space of Disse may interfere with immunosurveillance [53]. Furthermore, in both humans and mice the presence of lipogranulomas containing CD44^+^-macrophages accompanies the expansion of CD4^+^/FOXp3^+^ Tregs and tumor burden, suggesting a possible contribution of NAMs in promoting an immunosuppressive microenvironment [52] (Figure 1).

Interestingly, the tumor microenvironment of human HCCs is also abundantly infiltrated by TREM2^+^-macrophages displaying a pro-angiogenic and immunosuppressive phenotype [54] (Figure 1), while HCC patients with an expanded fraction of TREM2^+^-macrophages show poor survival rate [54]. Although these results suggest that TREM2 signaling might have a key role in liver carcinogenesis, the contribution of TREM2-expressing macrophages to HCC development still needs further investigation since the size and number of tumors is increased in TREM2-deficient mice subjected to hepatocarcinogenesis protocol [55]. This paradox might be explained considering the role of TREM2 in damping liver inflammation by switching macrophages from a pro-inflammatory to a tissue reparative phenotype [56]. Furthermore, it is possible that TREM2 liver macrophages might be more heterogeneous than what it emerges from present characterizations. The involvement of TREM2 in the setting of NAFLD/NASH is further supported by the recent demonstration that TREM2 plasma levels positively correlate with NASH severity in humans, pointing to the possible use of TREM2 as a diagnostic marker for patient stratification [57].

## The emerging role of neutrophil granulocytes in NASH-associated HCC

Following KCs activation aside from monocytes, neutrophils are rapidly recruited within the liver in response to CXCL1/2 and CXCL8 production leading to a significant expansion of hepatic neutrophil pool in NASH [28]. Neutrophil activation by DAMPs and PAMPs contributes to hepatic inflammation by ROS production and the release of cyto/chemokines, elastase, and myeloperoxidase (MPO) [58]. Elastase is also implicated in triggering NETosis, the process in which neutrophils produce extracellular traps (NETs), namely web-like structure consisting of DNA filaments, histones, and neutrophil proteases [58]. NETosis has been originally described as a mechanism for entrapping and killing microorganisms during infections [59]. However, several studies highlighted an increase in NET formation even in sterile conditions as in NASH, wherein it sustains inflammation and promotes HCC development [60]. This latter effect is facilitated by neutrophil interaction with activated platelets [61]. Along with that, neutrophil depletion significantly ameliorates pathological hallmarks associated with experimental NASH [62].

Several reports have also described significant neutrophil recruitment in different models of NASH-HCC, suggesting their contribution to cancer development [40,60,63]. Neutrophils are a heterogeneous cell population comprising several functional phenotypes among which those termed N1 and N2 are those better characterized [63]. N1 neutrophils show a cytotoxic activity toward cancer cells and are considered antitumor cells, while the N2 subset has protumor capability because of its immunosuppressive action against T-cells [63,64]. Neutrophils present in NASH livers and in NASH-HCC microenvironment produce high amount of TGF-β1 and acquire a N2-like phenotype that favour cancer escaping immunosurveillance [65]. Moreover, recent studies have evidenced further heterogeneity in tumor-associated neutrophils (TANs) isolated from NASH-HCC showing that these cells are specifically characterized by high expression of the chemokine receptor CXCR2 and the carcinoembryonic antigen-related cell adhesion molecule 8 (CEACAM8; CD66b) [66]. TANs in NASH-HCCs also release NETs that, through the engagement of Toll-like receptor 4 (TLR4), mediates a reprogramming of naïve CD4^+^ T-cells to CD4^+^/FOXp3^+^ Tregs capable of counteracting CD8^+^ T-cell functions [40] (Figure 1). This brings out the cross-talk between innate and adaptive immunity as a key mediator in NASH-associated tumorigenesis pointing out its potential use as a therapeutic target.

## The contribution of natural killer and natural killer T-cells to NASH-associated HCC

Natural killer (NK) cells are a heterogeneous component of innate immunity and in both mice and humans make up a large proportion of hepatic leukocytes [67,68]. Despite NK cells are abundant in the liver, up to now, their pathological implication in NASH is still matter of debate due to controversial results [69]. Recently, Wang et al. [70] demonstrate that NASH is accompanied by an expansion of activated NK cells characterized by high expression of the killer activating receptor NKG2D, granzyme B and INF-γ. These results are consistent with observations in human NASH showing increased levels of NKG2D in peripheral NK cells [71]. The contribution of NK cells to NASH is further corroborated by experiments using mice knockout for the transcription factor Nfil3 (Nfil3^−/−^) which lack conventional NK (cNK) cells without losing the fraction of liver resident NK (LrNK) cells [72]. As compared with wild-type littermates, Nfil3^−/−^ mice show an attenuation of the main features of NASH. On the contrary, NKp46-deficient mice lacking both cNK and LrNK cells are not protected from NASH-induced liver injury [73]. Altogether these results suggest that cNK cells might contribute to the disease worsening, whereas LrNK cells might have a protective action in NASH. So far, little is known about NK cell implications in NASH-HCC because of the limited number of studies. Nonetheless, data from HCC of viral etiology recently demonstrate that cNK and LrNK cells are reduced in the tumour microenvironment [74]. Furthermore, both subsets have a dysregulated phenotype characterized by an abundant expression of the checkpoint receptor T-cell immunoglobulin and mucin domain-containing protein 3 (TIM-3) that suppresses cytokine secretion and cytotoxicity hampering NK-mediated tumor surveillance [74] (Figure 1).

Natural Killer T (NKT) cells represent a particular subset of T-lymphocytes at the interface between innate and adaptive immunity. NKT cells are characterized by the co-expression of T-cell receptor (TCR) and the NK cell surface receptors (NK1.1 in mice or CD161/CD56 humans) [75]. The majority of hepatic NKT cells consists of type I or invariant NKT (iNKT), that use an invariant TCR encoded by Vα gene, while the remaining 5% is represented type II NKT cells relying instead on oligoclonal TCR repertoire [75]. NKT cells recognize lipid antigens presented by the MHC class I-like protein CD1d and are considered key players in mediating immune responses and tumor surveillance [75]. Available evidence indicates that NKT cells expand in NASH livers and plays a critical in the disease pathogenesis [75–77]. Indeed, interfering with NKT cells effectively improves parenchymal injury, inflammation, and fibrosis in different experimental models of NASH [76,78]. In particular, the lack of iNKT cells in *Jα18^−/−^* mice or the block of iNKT cells with retinoic acid receptor-γ agonist tazarotene reduces CD8^+^ T-cell infiltration in NASH livers [78], suggesting a strict interplay between cytotoxic T-cells and iNKT cells in the mechanisms supporting steatohepatitis. The combined depletion of CD8^+^ T-lymphocytes and NKT cells also prevents HCC development in *β2m^−/−^* mice receiving a choline-deficient high-fat diet [12]. Such an effect involves the production of the cytokine LIGHT, also known as tumor necrosis factor superfamily member 14 (TNFSF14), and LIGHT deficiency prevented HCC development without affecting the number or the activation status of CD8^+^ T cells but significantly reducing intra-hepatic NKT cell infiltration [12].

## Adaptive immunity in NASH progression to HCC: the role of B lymphocytes

As mentioned above, growing evidence points to the contribution of adaptive immunity in supporting lobular inflammation in NASH. In these settings, B-cell activation appears an early event in NASH [35,37], as the onset of experimental NASH is accompanied by a significant expansion of the fraction of CD43^−^/CD23^+^ B2-cells that contribute to hepatic inflammation by producing pro-inflammatory cytokines such as TNF-α and IL-6. B2-cell activation is fostered by the up-regulation in the hepatic expression of B-cell activating factor (BAFF) [35], one of the cytokines driving B-cell survival and maturation. Interestingly, circulating levels of BAFF are higher in patients with NASH than in those with simple steatosis and correlate with the severity of steatohepatitis and fibrosis [79]. Concerning the mechanism involved in triggering B-lymphocyte responses in NASH, recent data indicate the activation of both Myeloid Differentiation primary response 88 (MyD88) and B-cell receptor (BCR) signal pathways in response to gut dysbiosis. Indeed, fecal microbiota transplantation from NAFLD patients to healthy mice induces histopathologic hallmarks of the disease including an increased number of intrahepatic B-cells displaying an up-regulated expression of antigen-presenting and costimulatory molecules [37]. Beyond gut dysbiosis, oxidative stress can represent another important trigger for B-cell activation, since in NASH patients the prevalence of intrahepatic B/T cell aggregates associates with a higher level of circulating IgGs against oxidative stress-derived epitopes (OSEs) [35]. Furthermore, subcutaneous immunization with OSEs worsens experimental NASH by promoting a significant humoral reactivity along with an expansion of IFN-γ-producing CD4^+^ T helper cells [80]. In line with this, NASH microenvironment increases the antigen-presenting capability of B2-cells by stimulating the expression of the major histocompatibility complexes I (MHCI) and II (MHCII) and of the CD86 costimulatory molecule [37]. Moreover, *in vitro* intrahepatic B-cells directly influence CD4^+^ T helper (Th) cell functions by promoting Th1 activation [81]. Such an effect is mediated by intrahepatic, but not splenic B-cells, suggesting the presence of a peculiar B-cell phenotype differentiated locally without systemic involvement [37]. A cross-talk between B- and T-cells is further supported by data obtained in mice lacking B-cells or harboring functionally defective B-cells which show milder hepatic injury and lower liver recruitment of Th1-activated INF-γ^+^CD4^+^ T-cells as compared with control mice [35,37]. Furthermore, in NASH livers B-cells express pro-fibrogenic genes such as TGF-β1 and Timp-2, corroborating the already available data concerning the role of B-cells in tissue scarring [82–84]. It is noteworthy that NASH patients often show elevated circulating levels of IgA, which positively correlate with the fibrosis score [85]. At present the origin of these IgA is unknown. However, IgA-producing plasma cells are detectable in the livers of mice carrying the hepatocyte-specific deficiency for T Cell Protein Tyrosine Phosphatase (TCPTP) (AlbCrePtpn2fl/fl) that develop extensive NASH when fed with a high-fat diet [86].

Beside their role in promoting chronic inflammation and fibrogenesis, B-cells also display a pro-carcinogenic action [84]. Indeed, the amount of liver infiltrating B-cells in patients carrying HCC correlates with tumour aggressiveness and a shorter disease-free survival [84]. Single-cell-RNA-sequencing (scRNA-seq) also reveals that HCC matrix is enriched with plasma cells as compared with the hepatic tissue of cirrhotic or healthy donors [87], while HCC patients with a lower proportion of liver plasma cells display a higher survival rate [87]. Shalapour and colleagues have recently reported that IgA^+^ plasma cells associated with NASH-derived HCC have an immunosuppressive phenotype characterized by the expression of PD-L1 and IL-10 and that they inhibit CD8^+^ T-cell activation [86]. Along with that, IgA deficiency restores CD8^+^ T-cell capability to counteract tumour growth [87]. Overall, these data suggest the possibility that B-cell differentiation to IgA-secreting plasma cells may have a causative role in the development NASH-related HCC (Figure 1).

## Adaptive immunity in NASH progression to HCC: the role of T-cells

The possible implication of T-cells in NASH has been suggested by the high prevalence of IFN-γ producing Th1 CD4^+^ T-cells in both pediatric and adult NASH patients [88,89]. Moreover, INF-γ-deficient mice exposed to a steatogenic diet are lesser prone to develop extensive hepatic injury as compared to the wild-type counterpart [90]. Similarly, selective CD4^+^ T-cell depletion ameliorated hepatocellular injury by lowering the expression of IFN-γ along with classical-activated macrophage markers in OSE-immunized NASH mice [80]. These results are further corroborated by data obtained in humanized mice engrafted with a functional human immune system in which NASH development is accompanied by an expansion of CD4^+^ T-cells localized in the fibrotic regions and by an increased production of INF-γ and IL-17A. Of note, in the same mice, CD4^+^ T-cell depletion decreases NASH-associated immune cell infiltration, fibrosis, and overproduction of inflammatory mediators [91]. These latter observations would also imply a possible contribution of Th17 CD4^+^ T-cells that are characterized by the secretion of IL-17A. Although several studies have supported the implication of Th17 cells in NASH pathogenesis, the overall picture remains confused [92–96]. Recently, a single-cell RNA-sequencing (scRNA-seq) analysis has identified a NASH specific subset of hepatic Th17 cells named ihTh17 [97]. These cells are characterized by a high expression of C-X-C Motif Chemokine Receptor 3 (CXCR3) and by the secretion of large amounts of inflammatory mediators [97]. Of note, the hepatic accumulation of ihTh17 correlates with the extent of hepatocellular damage in both experimental and human NASH [97], while the adoptive transfer of ihTh17 cells induces hepatic injury in immunocompromised Rag2^−/−^ mice receiving high-fat diet which are normally protected from NASH [98]. Furthermore, Th17 cells also play a direct role in hepatic fibrogenesis as they can favor hepatic stellate cell (HSC) activation since, upon stimulation with IL-17A, HSCs become more responsive to the transforming growth factor-β (TGF-β), acquiring an increased capability of producing collagen fibers [99].

Beside CD4^+^ T-cells, mounting evidence points out the pathogenetic role of cytotoxic CD8^+^ T-cells in NASH. These cells are increased in both human and mouse NAFLD/NASH livers [12,80,100,101] and particularly adult NASH patients displaying a fibrosis score ≥2, suggesting a possible involvement in hepatic fibrogenesis [102]. The pathological implications of CD8^+^ T-cells in NASH is further corroborated by the observation that mice with an impaired CD8^+^ T-cell activation develop less steatosis and fibrosis as compared with control littermates [103]. Similarly, CD8^+^ T-cell depletion improves lobular inflammation and fibrosis by lowering the fraction of recruited macrophages and activated HSCs [12,101,104]. These experimental observations can be easily explained considering that liver recruited CD8^+^ T-cells display an activated phenotype characterized by an increased expression of pro-inflammatory mediators [12,105]. Notably, the hepatic recruitment and activation of CD8^+^ T-cells appears closely related to type I interferon signalling as chimeric mice lacking interferon α-receptor 1 subunit (INFα-R1) on CD8^+^ T-cells show lower hepatic recruitment of CD8^+^ T-cells. [105]. Recently, Dudek et al. [100] have characterized NASH-associated CD8^+^ T-cells showing that these cells feature C-X-C motif chemokine receptor 6 (CXCR6), effector molecules and the programmed cell death protein 1 (PD-1), this latter suggesting an activated/exhausted phenotype. Noteworthy, these authors have also reported that CXCR6^+^/PD1^+^/CD8^+^ T cells have an ‘auto-aggressive’ behaviour and, upon exposure to metabolic stimuli such as acetate and extracellular ATP, kill hepatocytes in an antigen-independent fashion. Overall, these data would suggest a critical role of CD8^+^ T-cells in perpetuating hepatic injury in NASH leading to tissue scarring. However, it has been recently proposed that tissue-resident memory CD8^+^ T (CD8^+^ Trm) cells have a role in controlling liver fibrosis during the resolution of experimental NASH. This effect depends on CD8^+^ Trm cell cytotoxic activity towards HSCs. Indeed, CD8^+^ Trm cells attract HSCs in a CCR5-dependent manner and stimulate their death through the Fas/Fas-ligand pathway [106]. These observations are further supported by data obtained in humans, where the liver accumulation of CD8^+^ Trm cells parallels the severity of the disease, suggesting a possible role of these cells in regulating NASH progression [106]. Considering these results, the scenario appears more complicated than the simple involvement of CD8^+^ T-cells in supporting disease progression. Therefore, further investigations need to define the precise role of distinct CD8^+^ T cell subsets in the different disease stages.

In parallel with the notions involving T-lymphocytes in the pathogenesis of NASH, increasing evidence point to their possible involvement in the process leading to HCC development. In this setting, multidimensional flow cytometry analysis of human HCC-infiltrating lymphocytes reveals an enrichment of CD4^+^ T-cells [107] which involve unconventional activated cells expressing both activation markers, such as the inducible co-stimulatory molecule (ICOS), and inhibitory receptors such as T-cell immunoreceptor with Ig and ITIM domains (TIGIT) and PD-1. Interestingly, these cells do not produce pro-inflammatory cytokines upon stimulation *ex vivo* but are instead characterized by the expression of the transcription factor Foxp3 and the proliferation marker Ki67, indicating that HCC development is associated with the expansion of locally proliferating regulatory T-cells (Tregs) [107] (Figure 1). These results are consistent with the concept that premalignant stages of NASH associate with a progressive hepatic accumulation of CD4^+^/Foxp3^+^ Tregs [40]. Tregs represent an immunosuppressive subset of CD4^+^ T-cells that specifically counteract T-cells functions, thus contributing to the loss of cancer immunosurveillance [108,109] (Figure 1). Consistently, Treg depletion in an experimental model of NASH-related HCC significantly limits tumour burden by increasing the hepatic abundance of INF-γ-producing CD4^+^ and CD8^+^ T-cells [40]. Beside Tregs, recent evidence points to a role for Th17 T-cells in the development of NASH-related HCC since pharmacological suppression of Th17 cell differentiation prevented HCC in NASH mice [13]. Interestingly, similar results have also been obtained by interfering with IL-17A signaling [13], indicating a possible therapeutic target for preventing NASH-associated HCC. These actions of CD4^+^ Tregs and Th17 cells are in contrast with the data showing that selective CD4^+^ T-cell depletion accelerates HCC growth when NASH is induced in mice with hepatocyte-specific over-expression of Myc [110]. In these animals, CD4^+^ T-cells loss results from mitochondrial oxidative stress consequent to disrupted lipid metabolism [110]. At present, it is unclear how these data relate with the expansion of CD4^+^ T-cells observed in many different models of NASH [33] and how CD4^+^ T-cells depletion can favour tumour growth.

Further conflicting data have been obtained in relation to the role of cytotoxic CD8^+^ T-lymphocytes in NASH-related HCC. In many experimental systems CD8^+^ T-cell depletion limits the incidence of tumours [41]. However, CD8^+^ T-cell ablation promotes HCC when transgenic mice over-expressing the urokinase plasminogen activator (uPA) are fed with a high-fat diet [86]. These discrepancies might be due to differences in the experimental settings as well as to the dual role played by these cells in supporting inflammation as well as in controlling cancer cell growth. [111]. A recent report from Pfister and co-workers [41] sheds some light on these inconsistencies by showing that depleting CD8^+^ T-cells following the onset of NASH, but prior to HCC development, effectively reduces HCC incidence in mice. Single-cell mapping of CD8^+^ T-cells has shown that they express activation/exhaustion markers and high levels of the immunomodulating molecule PD-1. Surprisingly, despite the high prevalence of CD8^+^/PD-1^+^ T-cells in NASH-driven HCCs these tumours do not respond to anti-PD-1 therapy which instead promotes an earlier HCC onset. A similar behaviour is also evident by inducing HCC on NASH background in PD1-deficient mice [41]. These observations are in contrast with previous reports showing the effectiveness of anti-PD-1 agents in promoting tumour regression in non-NAFLD HCC models [112] and suggest the possibility that in NASH-derived HCCs CD8^+^/PD-1^+^ T-cells lack immune-surveillance functions and have instead a tissue-damaging action, which is partially counteracted by PD-1 signalling, thus explaining the unfavourable effects of anti-PD-1 agents on tumour development [41] (Figure 1). Interestingly CD8^+^/PD-1^+^ T-cells with a gene expression profile comparable to that observed in rodent NASH are also detectable in human NAFLD/NASH livers suggesting the possibility that liver steatosis/steatohepatitis specifically activates CD8^+^/PD-1^+^ T-cells in a manner that favours the disease evolution and limits the response to HCC immunotherapy [41] (Figure 1). These observations are consistent with two recent meta-analysis considering eleven phase III clinical trials including more than 5,700 patients with advanced HCC which show that the benefits of immunotherapy targeting PD-1 or PDL-1 are mainly seen in patients with HCC of viral origin, whereas the same treatment is ineffective in patients with NAFLD-associated HCCs [41,113]. Along this view, Leslie and colleagues have recently reported that antagonizing CXCR2 in neutrophils sensitizes mice harbouring NASH-HCC to anti-PD-1 immunotherapy reducing the tumor burden and increasing the survival rate [66]. Such an effect appears related to a reprogramming of tumour granulocytes towards pro-inflammatory phenotype accompanied by an increased activation of XCR1^+^conventional type 1 dendritic cells (cDC1) and CD8^+^ T cells [66]. Whether confirmed in humans, the combined use of CXCR2/PD-1 inhibitors might represent a successful strategy to improve NASH-related HCC therapy by restoring cancer immunosurveillance. Nonetheless, additional studies using a more specific patient stratification for NAFLD/NASH etiology are urgently needed to better characterize the factors contributing to the poor response to current therapies in these subjects.

## Immunomodulating mechanisms underlying NASH-related HCC

**Figure 2:**
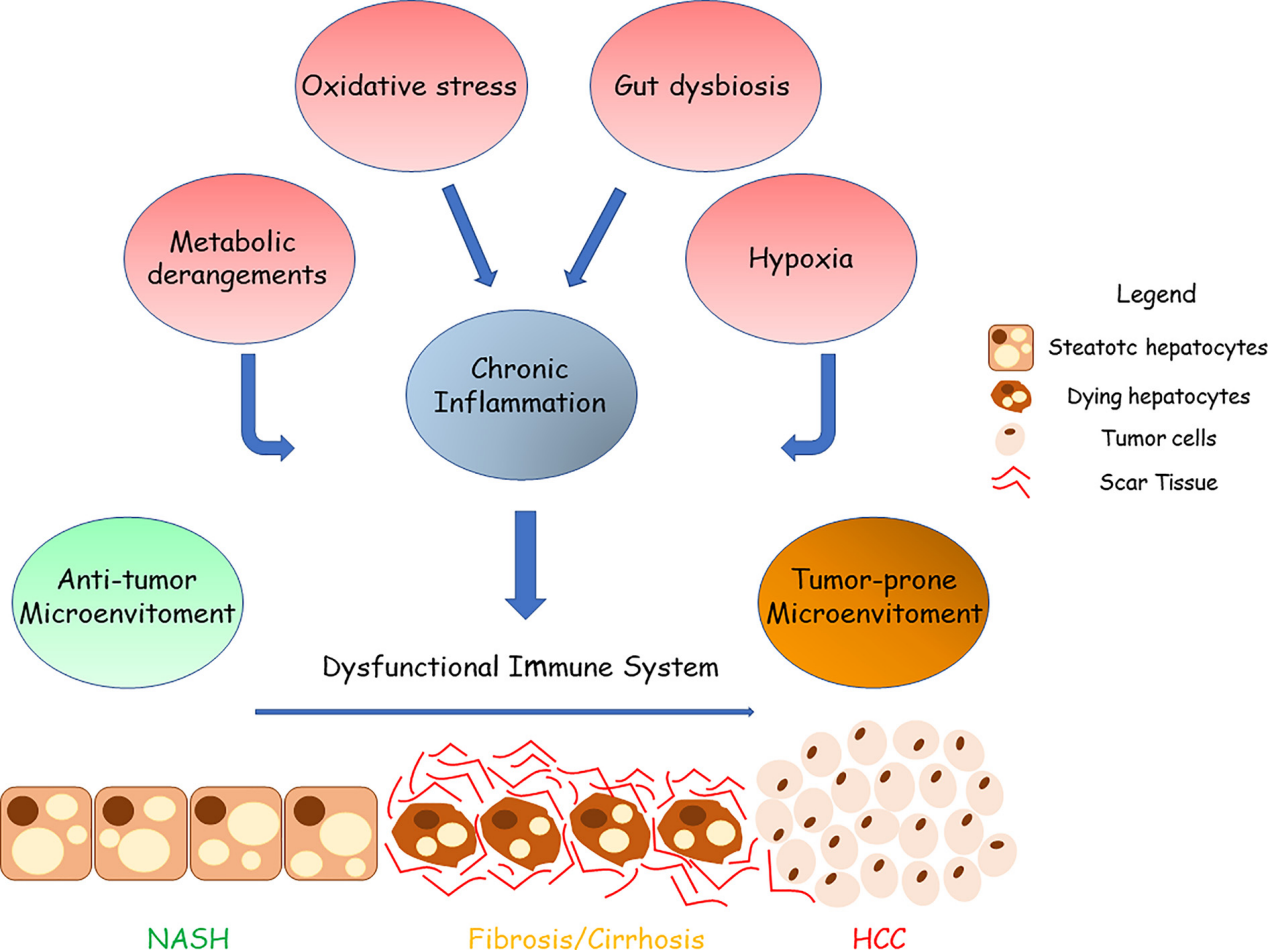
Contribution of chronic inflammation, metabolic imbalances, and hypoxia in reshaping the immune landscape in NASH-related HCC The transition from NASH to HCC is a complex process involving multiple factors such as lipotoxicity, oxidative stress, gut dysbiosis metabolic imbalances, chronic injury, and hypoxia that, in turn, stimulate chronic inflammation causing tissue scarring, and HCC development. Chronic inflammation, hypoxia and metabolic imbalances also induce a profound reprogramming of the immune system that results in the loss of its antitumour action, thus leading to a cancer-prone microenvironment in which malignant cells can proliferate undisturbed.

The data emerging from recent studies investigating the role of inflammatory and immune cells in the processes leading to NAFLD/NASH evolution to HCC have evidenced that the metabolic derangements associated with the disease evolution not only promotes liver inflammation but can also specifically influence the capacity of the immune system to counteract cancer cell growth. Indeed, NASH livers represent a unique biological microenvironment in which the coexistence of metabolic disorders along with chronic inflammation determine a significant reprogramming of the immune system [114] (Figure 2). In this context, the chronic activation of the immune cells stimulates the acquisition of an anti-inflammatory and/or exhausted phenotype leading to loss of immune surveillance and cancer development [114,115] (Figure 2). For instance, in patients with NASH-related HCC the prevalence of CD4^+^ and CD8^+^ expressing the inhibitory receptor Cytotoxic T-Lymphocyte Antigen 4 (CTLA-4; CD152) is higher as compared with those suffering from viral-related HCC [116]. Notably, the presence of CTLA-4^+^/CD8^+^ T cells positively correlates with serum palmitoleic acid (C16:1n7) to palmitic (C16:0) acid ratio, while *in vitro* CD8^+^ T-cells exposure to palmitic acid significantly enhances the fraction of CTLA-4 expressing cells [116]. CD8^+^ T cells from NASH-HCC also display impairment of multiple metabolic pathways such as glycolysis, fatty oxidation, and mitochondrial respiration. Such metabolic derangements result in altered cell motility that ultimately leads to the loss of anti-tumour capacity [117]. Of note, metformin supplementation restores CD8^+^ T cell functional properties by acting on cellular energy metabolism [117,118].

Among the metabolic imbalances occurring in NASH, aberrant cholesterol metabolism represents a typical hallmark with serious consequences for the disease evolution. In a recent study, Tang et al. [119] have reported that cholesterol accumulation within NKT cells induces lipid peroxidation leading to their functional impairment and favours orthotopic tumour growth in obese mice. In the same settings, the cytolytic functions of hepatic NKT cells are restored by treating mice with statins to normalize plasma cholesterol levels [120]. Overall, these results suggest that excessive cholesterol intake might favour cancer immune escape by affecting NKT functions during NASH progression (Figure 2). Therefore, strategies devoted to improving NKT cell-mediated immune responses might represent a possible therapeutic approach to control HCC development in NASH. Cholesterol accumulation also fosters the differentiation of dysfunctional restorative macrophages which persists even after cholesterol restriction [121]. Metabolic factors in combination with chronic antigen stimulation also impact on T-cells by favoring the hepatic accumulation of CD4^+^ and CD8^+^ T-cells expressing the thymocyte selection associated high mobility group box protein (TOX), a nuclear factor leading to the acquisition of an exhausted phenotype. Notably, TOX expression in effector T-cells promotes the up-regulation of inhibitory receptors such as PD-1 [115]. Concomitantly, NASH fosters the hepatic enrichment of tumour-associated macrophages (TAMs) displaying a high expression of PD-L1 that by binding to PD-1 on effector T cells transduces immunosuppressive signals [122] (Figure 2).

Beside the effects on lymphocytes, NASH liver environment has been proposed to favor the recruitment of myeloid-derived suppressor cells (MDSCs) [123]. MDSCs are a heterogeneous population of immature myeloid cells showing a variable differentiation state. MDSCs consist of two main subpopulations known as monocytic-MDSCs (M-MDSCs) and granulocytic or polymorphonuclear MDSCs (PMN-MDSCs). M-MDSCs share common phenotypic features with monocytes while PMN-MDSCs with neutrophils [124]. Regardless their subclassification, MDSCs play a critical role in tumour microenvironment (TME) by exerting immunosuppressive functions through the production of several factors including arginase 1 (Arg1), inducible nitric oxide synthase (iNOS), indoleamine 2,3-dioxygenase 1 (IDO1), TGFβ, IL-10, and ROS [124]. In addition, by secreting vascular endothelial growth factor (VEGF), prokineticin 2 (Prok2) and matrix metalloproteinase 9 (MMP9) MDSCs foster tissue remodelling and angiogenesis thus sustaining tumour growth. Interestingly, MDSCs are increased in NASH livers as well as in NASH-HCC [123,125] since low-grade inflammation and metabolic derangements stimulate the activation of the cell cycle-related kinase (CCRK)/mammalian target of the rapamycin complex 1 (mTORC1) that enhances the recruitment of MDSCs (Figure 1).

Finally, chronic tissue injury in NASH is strongly associated with a profound extracellular matrix remodeling resulting in liver fibrosis and cirrhosis which is a fertile ground for cancer development [16]. In this complex process, multiple factors are involved and, among them TGF-β1 which plays a critical role by supporting tissue scarring but also has a robust immunomodulatory action [126]. In this perspective, TGF-β1 stimulates functional changes in neutrophils that acquire an immunosuppressive phenotype [65] and can promote Treg differentiation [40] (Figure 2). As a result, the Treg expansion within tumours inhibits the functional activity of effector T-cells [40]. TGF-β1 also favours macrophage switching toward an anti-inflammatory phenotype that characterize tumour-associate macrophages (TAMs) [126]. Finally, TGF-β1 contributes to B-lymphocyte isotypic switch from IgM- to IgA-producing cells that, as mentioned above, are characterized by an immune-regulatory activity [86,127]. IgA^+^-producing plasma cells express inhibitory molecules such as PDL-1 and secrete IL-10, a potent anti-inflammatory cytokine that strongly inhibits cytotoxic CD8^+^ T cell function [128], thus impairing immune cancer surveillance in NASH-related HCC [86] (Figure 2).

Altogether these data strongly indicate that because of metabolic derangements that characterize the evolution of NAFLD/NASH the immune/inflammatory reactions associated with HCC development might greatly differ from those present in HCC of other etiologies possible explaining the clinical peculiarities of these specific tumors.

## Hypoxia inducible factors (HIFs) and NASH-related HCC inflammatory microenvironment

The previous sections have outlined the complexity of the factors influencing hepatic inflammation during NASH-related carcinogenesis. An additional issue is represented by the role played in NASH progression and HCC development by liver hypoxia and hypoxia inducible factors (HIFs) [129–135] (Figure 2). HIFs are a group of evolutionary conserved heterodimeric transcription factors, members of the basic helix-loop-helix Per-Arnt-Sim (bHLH-PAS) family, that respond to changes in cellular pO_2_ by regulating the expression of hundreds of HIF-target genes [136–139]. HIFs are formed by an α subunit (HIFα), which is hypoxia-inducible and oxygen-sensitive, and a constitutively expressed β subunit (HIFβ). Oxygen sensing relays on dioxygenases responsible for hydroxylating specific prolyl (prolyl-hydroxylases or PHDs) or asparaginyl (factor inhibiting HIF1 or FIH1) residues of HIFα subunit. Under normoxia, prolyl-hydroxylated HIFα is ubiquitinated by a E3 ubiquitin ligase complex which contains the von Hippel-Lindau (VHL) protein leading to proteasomal degradation. At the same time, HIFα hydroxylation on asparaginyl residues blocks the transcriptional activity of the heterodimer. Cell response to hypoxia is granted by the progressive inhibition of PHDs which are sensitive to modest decrease in pO_2_ and/or by blocking FIH1 activity, the latter sensitive to more severe hypoxic conditions [136–139]. The heterodimer HIF can then form a transcriptional complex with co-activator cAMP-response element binding (CREB)-binding protein (CBP) and histone acetyltransferase p300 (p300 HAT) which binds to hypoxia-responsive elements (HRE) in the promoter or enhancer sequences of target genes controlling: (i) the metabolic switch towards anaerobic glycolysis; (ii) intracellular pH regulation; (iii) angiogenesis and vasodilation; (iv) survival, proliferation, and stemness/differentiation; and (v) inflammatory responses [136–139].

Beside physiological functions, HIF-controlled target genes are also critical in the process of carcinogenesis in many organs including the liver. Indeed, a growing body of data points to the role of HIFs in regulating HCC angiogenesis, epithelial-to-mesenchymal transition, metastasis, and metabolic reprogramming [133–135,140,141]. In HCCs HIF target genes are activated not only by hypoxia but also by several different hypoxia-independent signals including growth factors, cytokines, metabolic or oxidative stresses, oncogene activation through ‘non-canonical regulation of HIF’ signal pathways [142,143]. These mechanisms might be relevant since, as recently discussed by Cramer and Vaupel [144], no reliable measurement of pO_2_ is so far available in human HCC although certain characteristics of these tumours such as hypervascularity, necrotic areas and resistance to therapy may suggest the presence of severe hypoxia in human HCC [144]. From the available data it emerges that HIF-1α activation may contribute to tumour development by stimulating cell proliferation, metabolic changes, angiogenesis, cancer invasion, and metastasis [145,146]. Conversely, the contribution of HIF-2α to HCC development is less well characterized [147–150] despite HIF-2α activation contributes to HCC resistance to chemotherapy [151]. Furthermore, *in vitro* data suggest that the knockdown of HIF-1α enhances the expression of HIF-2α and vice versa [152]. However, most of these results come from studies without indications of tumour aetiology or refers to virus-related HCCs.

The characterization of HIFs role in NAFLD/NASH has shown that HIF-2α regulated genes are involved in fatty acid synthesis/uptake and lipid storage [153,154]. Hepatocyte-specific HIF-2α deletion improves fatty liver, parenchymal injury, lobular inflammation in NASH ameliorating the disease evolution to fibrosis [155]. These results are consistent with the observation that HIF-2α activation is a key feature of human NAFLD correlating with the prevelence of fibrosis [154]. Moreover, we have reported that HIF-2α is over-expressed in two third of HCC developing in NAFLD patients with HIF-2α nuclear localization beeing prevalent in HCCs originating in cirrhotic livers [14]. The importance of HIF-2α is further supported by experiments inducing NASH-derived HCCs in mice defective for hepatocyte HIF-2α (hHIF-2α^−/−^ mice). In these animals, HIF-2α depletion halves the number and the size of HCC nodules as compared with wild-type mice [14]. Such an effect associates with an induction of p21 and p53 in cancer cells, indicating that hepatocyte HIF-2α can directly promote cancer cell proliferation and survival (Figure 1) [14]. The same experiments also show that lack of HIF-2α in parenchymal cells decreases HCC TAM infiltration as well as PDL-1 transcripts [14]. Although a study by Imtiyaz et al. [156] has shown that macrophage HIF2α modulates the expression of CXCR4, M-CSFR, and fibronectin 1 (FN1) favouring macrophage tumor infiltration, our data suggest a possible additional contribution of hepatocyte-derived factors in modulating TAM recruitment in NASH-derived HCCs. We have shown in fact that SerpinB3 (SB3), a HIF2α-regulated serine protease inhibitor, acts as a pro-inflammatory mediator in the progression of experimental NASH stimulating the infiltration of TREM-2^+^ macrophages and the up-regulation of pro-inflammatory cytokines [157]. Interestingly, in humans NASH-HCCs HIF-2α expression significantly correlates with that of SB3, while in experimental models the interference with hepatocyte HIF-2α affects SB3 expression [155]. However, SB3 is not the only HIF-2α-regulated mediator produced by hepatocytes as in NASH livers HIF-2α stimulates the secretion on the histidine rich glycoprotein (HRG) which plays a role in sustaining hepatic inflammation [155]. At present, the mechanisms by which SB3 might promote macrophage recruitment in HCC and the possible interplay between SB3 and HRG are still poorly characterized.

HIF1α might have additional roles in modulating TAM function in HCCs as Wu et al. [158] have reported that in HCC patients HIF1α stimulates the macrophage expression of the pro-inflammatory modulator Trigering Receptor Expressed on Myeloid cells-1 (TREM-1) and that TREM-1 positive TAMs recruit immunosuppressive Tregs to HCC, leading to both reduced infiltration of CD8^+^ T-cells and poor survival. Accordingly, the block of TREM-1 positive TAMs reverse immunosuppression and anti-PDL1 resistance in HCC [158]. Additional links relating HIF1α, NASH and HCC emerge from studies using mice partially defective for the SART1 gene which encodes for a novel oxygen-independent HIF-1α ubiquitin ligase, named hypoxia-associated factor (HAF), which is responsible for selective oxygen-independent degradation of HIF-1α [159]. Male SART1^+/−^ mice, spontaneously develop NASH-related HCC in association with a significant up-regulation of HIF-1α in either circulating and liver infiltrating immune cells, but not in hepatocytes. Macrophages from these mice show an increased production of the cytokine CCL5 leading to an increased infiltration of neutrophils and CCL5 neutralization decreases both neutrophil infiltration and HCC development in SART1^+/−^ [160]. These results are in line with the notion that HIF1α can sustain neutrophil survival under hypoxic conditions through NF-kB signalling [161] and that cancer cells can recruit these leukocytes by secreting several chemokines [162]. Interestingly, HIF1α may also contribute to N2 polarization of neutrophils [108], while antibody-mediated neutrophil depletion attenuates HCC development in mice [163]. More pertinent to NASH-related HCC the loss of nfkb1 promotes neutrophilia and an aging-related chronic liver damage associated with fatty liver, fibrosis, and HCC development [163].

MDSCs represent another cell population that may be potentially modulated by HIFs in HCC, since MDSCs have been detected mostly in hypoxic regions of human HCC [164]. Accordingly, HIF1α, has been reported to activate tumour-associated MDSCs resulting in the suppression of both antigen-specific and non-antigen-specific T-cells [165] through the expression of PD-L1 [166]. Nonetheless, the knowlwdge on relative roles of HIF-1 and HIF-2 in modulating inflammatory/immune cell function in NASH and NASH-related HCC still remains largely incomplete and future studies in this field are strongly deserved.

## Conclusion and perspectives

In recent years the contribution of specific immune/inflammatory mechanisms in supporting NAFLD/NASH evolution to HCC has received increasing interest and growing data ha ve shed light on the involvement of different subsets of myeloid and lymphocytic cells. Furthermore, emerging evidence indicates that these cells acquire peculiar phenotypes in the tumour environment that might strongly influence their behaviour during the disease progression and in the carcinogenesis process. The interplay between different liver cell populations is also strongly influenced by gut-derived bacterial products, metabolic/nutritional signals, and hypoxia. These factors are likely capable of modulating in opposite ways cellular responses, possibly explaining why immune/inflammatory cells can sustain HCC development by supporting chronic inflammation and at the same time favour cancer cell immune evasion. Understanding this complexity will represent the challenge for researchers in this field in the next years. The impact of these studies on the diagnosis and the treatment of NASH-related HCC is already appreciable, explaining possible reasons for the poor response to immune checkpoint inhibitors observed in these patients. From this, it is possible to forecast that more detailed information on the actions played by immune/inflammatory cells in NASH-associated carcinogenesis would lead to innovative therapies tailored on this form of HCC.

## Abbreviations

CBP: cAMP-response element binding-binding protein
CREB: cAMP-response element binding
EGF: endothelial growth factor
FGF: fibroblast growth factor
FN1: fibronectin 1
HAF: hypoxia-associated factor
HGF: hepatocyte growth factor
HRE: hypoxia-responsive elements
IGF-1: insulin-like growth factor
IL-6: interleukin-6
MDSC: myeloid-derived suppressor cell
mTORC1: mammalian target of the rapamycin complex 1
NAFLD: nonalcoholic fatty liver disease
PDGF: platelet-derived growth factor
SB3: SerpinB3
TAM: tumour-associated macrophage
TGF-β1: transforming growth factor β
TNFα: tumour necrosis factor α
VEGF: vascular endothelial growth factor
VHL: von Hippel-Lindau
